# The Adverse Impact of Tumor Microenvironment on NK-Cell

**DOI:** 10.3389/fimmu.2021.633361

**Published:** 2021-06-11

**Authors:** Ziming Hu, Xiuxiu Xu, Haiming Wei

**Affiliations:** ^1^ Hefei National Laboratory for Physical Sciences at Microscale, Division of Life Science and Medicine, University of Science and Technology of China, Heifei, China; ^2^ Institute of Immunology, University of Science and Technology of China, Heifei, China

**Keywords:** NK cells, tumor microenvironment, cytotoxicity, migration, metabolism

## Abstract

NK cells are considered an important component of innate immunity, which is the first line of defensing against tumors and viral infections in the absence of prior sensitization. NK cells express an array of germline-encoded receptors, which allow them to eliminate abnormal cells and were previously considered a homogenous population of innate lymphocytes, with limited phenotypic and functional diversity. Although their characteristics are related to their developmental origins, other factors, such as tumors and viral infections, can influence their phenotype. Here, we provide an overview of NK cells in the context of the tumor microenvironment, with a primary focus on their phenotypes, functions, and roles in tumor micro-environment. A comprehensive understanding of NK cells in the tumor microenvironment will provide a theoretical basis for the development of NK cell immunotherapy.

## Introduction

Natural killer (NK) cells, which originate in the bone marrow, were first identified in 1975 (1, 2). NK cells are generated from common lymphoid progenitor cells, which develop common innate lymphoid cell progenitors. The common innate lymphoid cell progenitors subsequently give rise to the NK-restricted NK cell progenitors. NK cells are defined as CD3^+^CD56^-^ lymphocytes, and are distinguished into CD56^bright^ and CD56^dim^ subsets. CD56^bright^ NK cells usually express CD122, NKp46, and NKp80, while CD56^dim^ NK cells express more markers, including CD16, CD57, and PEN5. NK cells can be found in peripheral, lymph node, spleen, liver, lung, and bone marrow (3–6). More than 90% of peripheral blood, lung, bone marrow, and spleen NK cells belong to the CD56^dim^CD16^+^ subset, which exhibit marked cytotoxic function on interaction with target cells. In contrast, most NK cells in lymph nodes belong to the CD56^bright^CD16^-^ subset, and have predominantly immune regulatory characteristics (7). When NK cells meet with stressed cells, they produce lytic granules, containing factors such as perforin, granzymes, and granulysin, which can induce cell death. Further, NK cells can induce apoptosis of target cells by binding to their FAS or TRAILR receptors. NK cells produce an array of cytokines [interferon (IFN)-γ, tumor necrosis factor (TNF)-α, interleukin (IL)-10], growth factors (granulocyte-macrophage colony-stimulating factor), and chemokines (CCL3, CCL4, CCL5, XCL1), and can shape immune responses through their interactions with dendritic cells (DCs), macrophages, and T cells (8–10).

The NK cell cytotoxic attack is immediate, and does not require prior antigen-priming or MHC-restriction, and NK cell status depends on the balance of activating and inhibitory signals among the various receptors interacting with their ligands (11). Activating receptors include the cytotoxicity receptors (NCRs; NKp46, NKp30, and NKp44), C-type lectin receptors (CD94/NKG2C, NKG2D, NKG2E/H, and NKG2F), and killer cell immunoglobulin-like receptors (KIRs) (KIR-2DS and KIR-3DS). Barrow and colleagues reported that natural killer cell p44-related protein (NKp44) can recognize platelet-derived growth factor-DD, which is produced by proliferating tumor cells and can activate NK cells (12). Inhibitory receptors include C-type lectin receptors (CD94/NKG2A/B) and KIRs (KIR-2DL and KIR-3DL). MHC class I (MHC-I) molecules are present on most cells, and NK cell inhibitory receptors (KIRs and CD94/NKG2A/B) can bind to them to prevent NK cell-mediated killing (13–17); however, when stressed cells downregulated MHC-I expression, NK cells are activated through “missing-self recognition” by losing their inhibitory signals. When cancer cells show elevated level of NK cell receptor, like NKG2D in response to stress, “self-induced” activation mechanism occur and leading the engagement of NK cells. Despite the expression of the inhibitory receptor, the activation of the “induced self” override the inhibitory signals present on cancer cells. These two mechanisms are not contradictory, and may co-regulated the overall response of NK cells to pathogens (18).

Carrega et al. showed that, in patients with non-small cell lung cancer, tumor-infiltrating NK cells express several activation markers, including NKp44, CD69, and HLA-DR, yet showed profoundly impaired cytotoxic potential (19). Further, an inverse correlation was demonstrated between circulating or tumor-infiltrating NK cell levels and the presence of metastases in patients with various types of solid tumours (20–22). In this review, we summarize recent developments and gaps in knowledge relating to tumor-infiltrated NK cells.

## Tumor-Infiltrated NK Cells Exhibit an Altered Phenotype

NK cells have been observed in many types of tumors, including primary tumors, metastases, and tumor-infiltrated lymph nodes (23–25). In non-small cell lung cancer, CD56^bright^ and CD16^-^ NK cells were observed in the tumor stroma. Although these NK cells displayed some activation markers, such as NKp44, CD69, and HLA-DR, their cytolytic potential was lower than that of NK cells in the peripheral blood and normal lung tissue (19). NK cells are rarely detected in colorectal carcinoma tissue; however, adjacent normal mucosa contained normal levels of NK cells (26). Besides, high levels of CD57^+^ NK cell infiltration is associated with good prognosis, while NKp46^+^ infiltrate has no prognostic value (27). Few NK cells are detected in endometrial tumors and tumor-resident CD103^+^ NK cells express more co-inhibitory molecules, such as TIGIT and TIM3, depending on the severity of the disease (28). Zhang et al. demonstrated that TIGIT is associated with NK cell exhaustion in tumor-bearing mice and patients with colon cancer, and blockade of TIGIT prevents NK cell exhaustion and promotes NK cell-dependent tumor immunity in several tumor-bearing mouse models (29). The tumor associated circulating NK from the patients of prostate cancer increased the expression of markers of exhaustion (PD-1, TIM-3) and were impaired in their degranulation capabilities (30). Izawa et al. found that the ratio of tumor infiltrating CD56^dim^ NK cells gradually decreased, according to disease progression, due to the relatively higher sensitivity of CD56^dim^ NK cells to apoptosis in response to H₂O₂ in the tumor microenvironment. Further, exposure of NK cells to H₂O₂ results in impaired antibody-dependent cellular cytotoxicity (31). Moreover, Carrega et al. reported that the percentage of NK cells was lower in neoplastic tissue than in equivalent normal tissue. These researchers also found that, in lung and breast cancer, levels of CD56^bright^ perforin^low^ NK cells were significantly higher than those in matched normal tissue (32). In addition, several studies have indicated that there is a correlation between high NK cell infiltration and better prognosis in renal cell carcinoma (33–36).

Natural Cytotoxicity Receptors (NCRs) include of NKp44, NKp46 and NKp30 and they can play an important role in most functions exerted by NK cells. NKp44 is a transmembrane glycoprotein and it has three mRNA splice variants which display different signaling capability. NKp44-1has the ITIM in their cytoplasmic tail but NKp44-2 and 3 are not. NKp44 ligands which expressed by tumor cells comprises cellular and cell-released forms. Mixed-lineage leukemia protein-5 (MLL5), termed 21spe-MLL5, Cell surface-associated heparan sulfate (HS) proteoglycans (HSPGs) and Proliferating Cell Nuclear Antigen (PCNA) are expressed on the surface of tumor cells, while Platelet-Derived Growth Factor (PDGF)-DD and Nidogen-1 (NID1) glycoprotein are secreted by tumor cells as soluble molecules to interact with NKp44. Among these NKp44 ligands, 21spe-MLL5, HSPG and (PDGF)-DD interact with NKp44 result in activation of NK cells. However, PCNA and NID1 inhibit the cytolytic function of NK cells. Tumor-infiltrated NK cells expressed higher level of KLRC1(NKG2A) gene and KLRD1(CD94). Also, KLRB1 gene (CD161) was expressed on tumor-infiltrated NK cells, which could bind to the CLE2D ligand on tumor cells to inhibit NK cell-mediated cytotoxicity (37–40). Small cell lung cancer primary tumors expressed very low level of NKG2DL mRNA and small cell lung cancer lines express little to no surface NKG2DL at the protein level, which caused the evading NK surveillance (41). Although the activating receptor NKG2D induces NK cell-mediated killing of metastasizing tumor cells by recognition of the stress-induced ligands MICA, MICB, and ULBP1-6. However, platelets enable escape from this immune surveillance mechanism by obstructing the interactions between NK cells and tumor cells or by cleaving the stress-induced ligands (42).

Anahid Jewett et al. recently indicated that NK cells could select and kill cancer stem cells/undifferentiated tumors. Cancer stem-like cells had a specific genetic signature and sustained tumor growth due to their self-renewal capacity. NK cells triggered differentiation of CSCs/undifferentiated tumors primarily *via* secreted and membrane bound forms of IFN-γ. Thus, NK cells played an important and unique role in targeting stem-like tumors or poorly differentiated tumors (43, 44). CD94, NKG2A, NKp46 and CD69 were considered as the phenotype as memory-like NK cells and the memory-like NK cells could be induced by IL-12, IL-15 and IL-18. Memory-like NK cells able to lyse autologous tumor cells can also be generated from patients with solid malignancies. The anti-tumor activity of allogenic and autologous memory-like NK cells is significantly greater than that displayed by NK cells stimulated overnight with IL-2. Also, memory-like NK cells displaying high levels of anti-tumor activity and low levels of reactivity against non-malignant cells, which could be transferred to future clinical trials of adoptive NK cell therapy (45).

Huergo Zapico et al. found that when NK cells were co-cultured with melanoma cells, melanoma cells up-regulated the expression of stem cell marker CD271 and CD166. In addition, melanoma cells showed cadherin switching, increased fibronectin expression and cytoskeletal recombination, indicating F-actin stress fiber production. Melamo cells could induce down regulation of NKp30, NKG2D and DNAM-1 on NK cells. However, the melanoma cell lines had little effect on the expression of Tim-3. Compared with regions far from NK cells, the expression of E-cadherin was lower in regions close to NK cells, and the expression of N-cadherin was higher in the region close to NK cells. These data clearly suggest that, at least in some cases, NK cells can influence the EMT at the tumor site (46).

Scavenger receptor MARCO, which is expressed on a specific subpopulation of TAMs in the tumor. When using anti-MARCO treatment to mouse model, the killing ability of NK cells were enhanced and the amount of IL-15 is the serum was also enhanced. The author indicated that IL-15 production induced by anti-MARCO locally in the tumor and possibly the draining lymph node will support the proliferation, migration, and cytotoxic capacity of NK cells (47). Sialic acids, extracellular matrix/collagen or aminophospholipids was expressed on the surface of tumor respectively, which could be recognized by sialic acids, extracellular matrix/collagen or aminophospholipids expressed on the surface of NK cells. Thus, NK cell function was inhibited (48).

## The Cytotoxicity of Tumor-Infiltrated NK Cells Is Impaired

**Figure 1:**
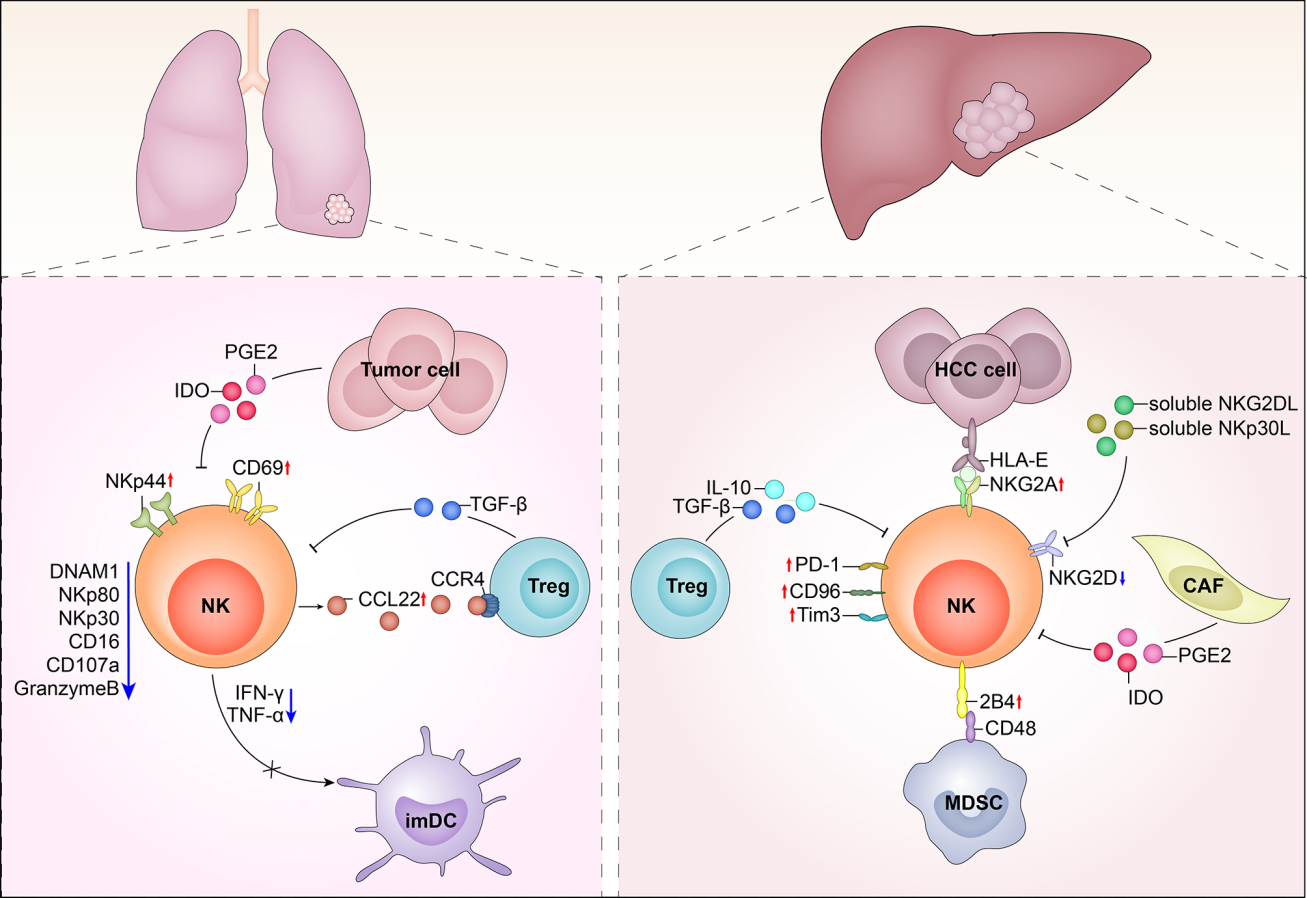
Tumor infiltrates NK cells in hepatocellular carcinoma (HCC) and lung cancer. Left image: In the tumor microenvironment of lung cancer, NK cells up-regulated the expressions of CD69 and NKp44 and down-regulated the expression of NKp30, NKp80, DNAM-1 and CD16. Series of soluble molecules were secreted by tumor cells, like IDO, PGE2, and TGF-β. Tumor cells also could express membrane molecules that can shed receptors on the surface of NK cells. In addition, intratumoral NK cells showed impaired IFN-γ secretion, which may lead to inefficiency in DC maturation. Right image: In the tumor microenvironment of HCC, NK cells up-regulated KIR, NKG2A, PD1, TIM2, and CD96 inhibitory receptors while down-regulated NKG2D. Treg cells produced IL-10 and TGF-β. In HCC, TGFβ, prostaglandin E2 (PGE2) and indoleamine 2,3-dioxygenase (IDO), produced by tumor-associated fibroblasts inhibit the cytotoxic activity and cytokine secretion of NK cells.

The tumor microenvironment is a complex milieu, full of inhibitory cells and factors. Tumor-associated macrophages (TAMs) accumulate at the tumor site, as well as circulating monocytes, recruited by the tumor-derived chemotactic factor, CCL2. Initially, these monocytes polarize into M1 cells, which exhibit cytotoxicity against tumor cells. M1 cells secrete cytokines, including IFN-γ and IL-12, which activate NK cells; however, with carcinoma progression and metastasis, TAMs polarize into cells and secrete large amounts of IL-10 and TGF-β, which suppress NK cell cytotoxicity. In contrast, M2 cells stimulate regulatory T cells (Tregs) and Th2 cells, which generate an immunosuppressive environment for NK cells (49). Other immunosuppressive cells within tumors are myeloid derived suppressive cells (MDSC), which include granulocytes, macrophages, and dendritic cells that are blocked at various stages of maturation MDSCs produce immunosuppressive factors such as IL-10, TGF-β, and IL-4. TGF-β inhibits the expression of two NK cell receptors, NKp30 and NKG2D, which are critical for tumor cell recognition and killing, as well as for functional interaction between NK cells and DC. IL-4 strongly reduces the ability of NK cells to kill sensitive targets and produce cytokine. Indoleamine 2,3-dioxygenase (IDO) promotes the production of the immunosuppressive tryptophan catabolite, L-kynurenine, which interferes with the IL-2-induced upregulation of NKp46 and NKG2D expression, thereby reducing the ability of NK cells to recognize and kill tumor cells (50–52). The expression of CD16 is down-regulated in most solid tumors-infiltrated NK cells, which may be related to the reduced proportion of CD56dim NK cells (32). CD57 is a marker of NK cell terminal differentiation, and CD57+ NK cells have high cytotoxic potential (53, 54). CD56^dim^KIR^+^CD57^+^ NK cells were observed in the peripheral blood of patients with acute myeloid leukemia (55). In addition, tumor-infiltrating NK cells in non-small-cell lung cancer and melanoma metastatic lymph nodes exhibit downregulation of the activation markers, CD69, NKp44, and HLA-DR (19, 56), while TIGIT, TIM-3, LAG-3, and PD-1 were upregulated in tumor-infiltrated NK cells, indicating that they tended toward exhaustion (57). In the tumor microenvironment CD49a^-^CD49b^+^Eomes^+^, NK cells can convert into CD49a^+^CD49b^+^Eomes^+^ and CD49a^+^CD49b^-^Eomes^int^ NK cells (type 1 innate lymphoid cells) in response to cytokine-TGFβ signaling; however, intermediate group 1 innate lymphoid cells and group 1 innate lymphoid cells could not control local tumor growth and metastasis, and TGF-β signaling in NKp46^+^ cells suppress NK cell-mediated tumor immunosurveillance (57) (**Figure 1**).

## NK Cell Metabolism Is Dysregulated by the Tumor Microenvironment

Cong et al. investigated the role of NK cells in the tumor microenvironment by using credibly induced KrasG12D(KRAS) knockout into mouse lung cancer models. Further, they applied anti-NK1.1 monoclonal antibody, PK136to depletion of number of NK cells, or Kras mice with an Nfil3-/- mouse knockout model, and showed that depletion of NK cells significantly accelerated tumor development during tumor initiation, while depletion of NK cells during promotion and development had no effect on tumor development. The author hypothesized that NK cells could effectively prevent the occurrence of tumor, but could not control the occurrence and development of lung cancer. In addition, they found that the quantity of NK cells, T cells, B cells, and MDSCs in the lungs declined progressively, while the number the macrophages were increased, particularly the quantity of M2 cells, which function as immune suppressive cells. Further, tumor-infiltrated NK cells showed significantly attenuated cytotoxicity, and the expression levels of granzyme B, perforin, CD107a, IFN-γ, and TNF-α were gradually reduced in lung NK cells during lung cancer development. In contrast, the expression of molecules associated with activation and cytotoxicity, including NKp46, CD69, CD44, CD226, CD16/32, FasL, TRAIL, and CD122, and the inhibitory molecules, CTLA4, CD96, CD94, PD-1, PD-L1, Tim3, CD276, LAG3, and CD244, were unchanged in lung NK cells during lung cancer development.

As established, glucose metabolism is essential for the function of human and mouse NK cells; hence, dysregulation of glucose metabolism can lead to NK cell dysfunction. FBP1 is important in glucose metabolism and inhibits glycolysis in human tumor cells and hematopoietic progenitor cells. Levels of FBP1 showed a 69-fold increase in tumor-infiltrated NK cells, in which glycolysis was inhibited; however, when FBP1 was inhibited, NK cell glycolysis function was restored. Thus, FBP1 weakens the cytotoxicity of tumor-infiltrated NK cells by inhibiting glycolysis; however, FBP1 can also impair the viability of tumor-infiltrated NK cells directly and independently of glycolysis. Lactic acid accumulated in the tumor microenvironment is a potent inhibitor of NK cell effector function and viability. Intracellular acidification and decreased ATP synthesis caused by lactic acid may be related to impaired IFN-γ production by NK cells (58).

**Figure 2:**
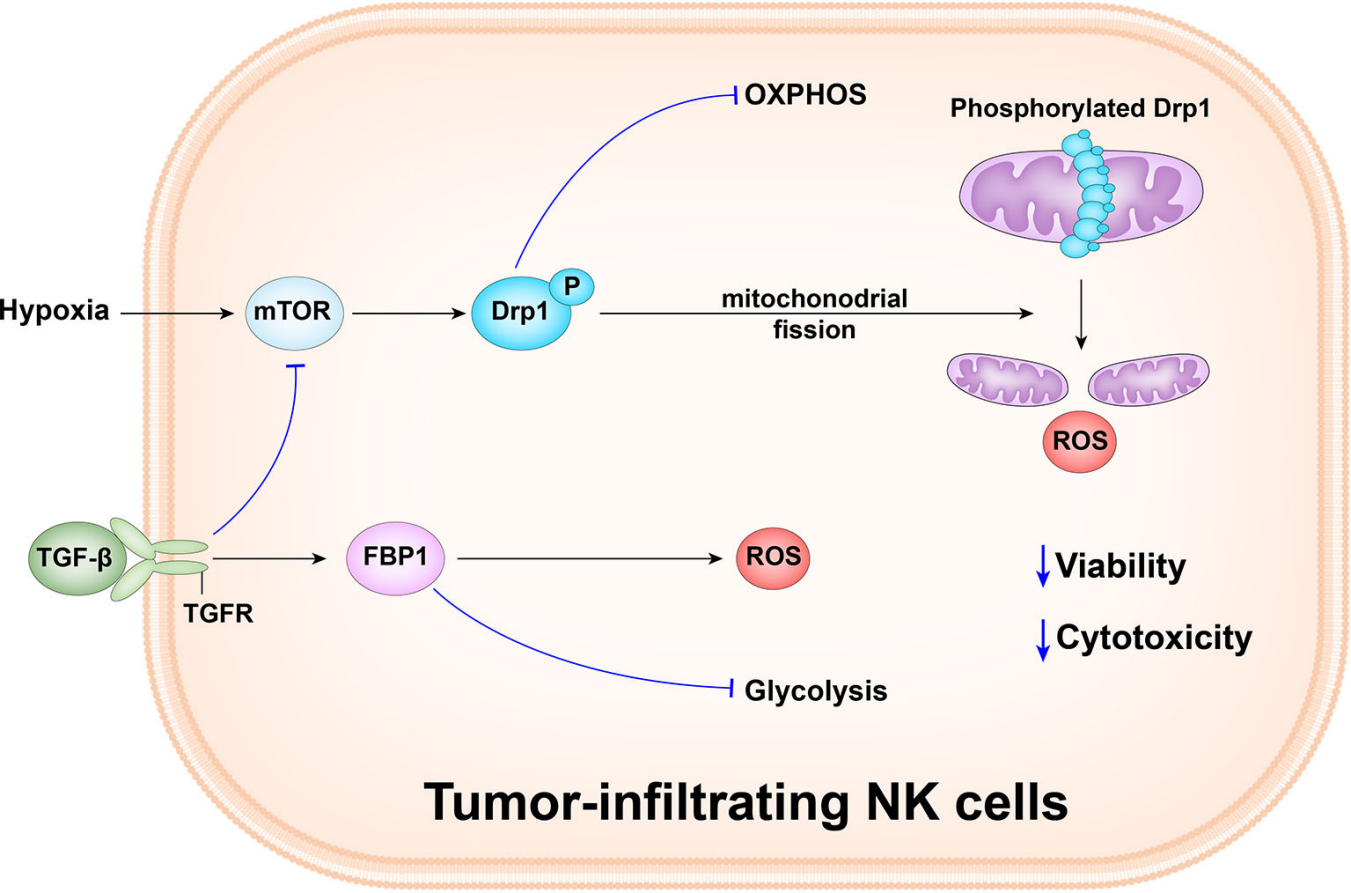
Tumor-infiltrating NK cells in human liver cancer have small, fragmented mitochondria in their cytoplasm, while tumor and peripheral NK cells in the liver have normal large, tubular mitochondria. Mitochondrial fragmentation causes tumor to evade NK cell-mediated surveillance. The hypoxic tumor microenvironment promotes the continuous activation of the mechanism target of rapamycin-GTPase motor associated protein 1(mTOR-DRP1) in NK cells, leading to excessive mitochondrial fragmentation and enhance the viability and anti-tumor ability of NK cells. Besides, the abnormally expressed gluconeogenic enzyme, FBP1, in response to TGFβ, can inhibit NK cell glycolysis and promotes the production of reactive oxygen species (ROS), and causes dysfunction by inhibiting glycolysis and reducing activity.

Zheng et al. observed that the tumor-infiltrated NK cells mainly had small, fragmented, distinct mitochondria in the cytoplasm, whereas liver and peripheral NK cells primarily had large, tubular, and densely packed mitochondria. There was a positive correlation between mitochondrial length and granzyme B levels. Furthermore, tumor-infiltrated NK cells had a significantly lower mitochondrial mass than paired tumor-adjacent normal liver NK cells. Moreover, the cells also had increased mitochondrial ROS levels and tumor infiltrated NK cells had upregulated expression of numerous mitochondrial fission-related genes, including *INF2*, *MIEF2*, *FIS1*, and *GDAP1*; high expression of fission genes drives mitochondrial fragmentation. Hypoxia is a key feature of the tumor microenvironment and tumor infiltrated NK cells are enriched for hypoxia signatures, including expression of HK2, SLC7A5, SLC2A3, and KDM3A. In addition, NK cells cultured under hypoxia show reduced expression of granzyme B, IFN-γ, and CD107a after activation, suggesting impaired functionality. Drp1 is the main regulator of mitochondrial fission, and drives division at specific points along mitochondria. Compared with paired tumor-adjacent normal liver NK cells, tumor-infiltrated NK cells upregulate Drp1 pro-fission activity through phosphorylation of its Ser616 residue. Restoration of mitochondrial morphology by knocking down Drp1, and in response to mdivi-1 treatment, enabled tumor-infiltrated NK cells to kill tumor cells. Therefore, mitochondrial fragmentation is correlated with decreased NK cell antitumor capacity (59) (**Figure 2**).

Obesity induces lipid accumulation driven by peroxisome proliferation-activated receptor (PPAR) in NK cells, resulting in complete “paralysis” of cell metabolism and transport, which reduces the anti-tumor response of NK cells and fails to reduce tumor growth *in vivo* experiment with obesity (55). Frank Cichocki et al. found that in adaptive NK cells, AT-rich interaction domain 5B (ARID5b), a short isoform of chromatin modified transcriptional regulator, was selectively induced by DNA hypopethylation. Knockdown and overexpression studies have shown that ARID5b plays a direct role in promoting mitochondrial membrane potential, gene expression encoding electron-transport chain components, oxidative metabolism, survival, and IFN-γ production (56). In addition, mTOR is sensitive to nutrient utilization and can be inhibited by TGF-β, thereby inhibiting NK cell metabolism and function. Therefore, it can be speculated that TGF-β production is also higher in nutrient-deficient TMEs, and mTOR may be inhibited, thereby limiting the effector function of NK cells (57). Studies by Antonie Marcaus et al. showed that after high concentration of IL-15 was exposed to NK cells, metabolic checkpoint kinase mTOR was activated and promoted bioenergy metabolism. This process is essential for maintaining the proliferation of NK cells during development and for achieving anti-tumor cell lysis (60).

Róisín M. Loftus et al. found that NK cells isolated from human solid tumors were deficient in their pro-inflammatory functions, including production of IFN-γ and tumor cytotoxicity. Tumor cells are known to have a high demand for glutamine in addition to glucose, so it is likely that low levels of glutamine are also present in the tumor microenvironment. Glutamine limited tumor microenvironment can inhibit the expression of cMyc in NK cells, resulting in reduced NK cell metabolism and inhibition of anti-tumor NK cell function (61, 62).

## Effects of the Tumor Microenvironment on NK Cell Migration

High NK-cell infiltration is often believed to be an indicator of better prognosis (28), and CD56^bright^ cells may be preferentially recruited tumor sites (19, 63); however, CD56^bright^ cells have been associated with poor cytolytic function. CCR5 is a receptor for MIP-1b, which is an adhesive signal that leads to the arrest of leukocytes within tissue. Several studies have indicated that only CD56^bright^ CD16^-^ peripheral blood NK cells express CCR5, which may explain their accumulation in tumor tissues. NK cells are not normally associated with secondary lymphoid organs. Indeed, CD16^+^ NK cells both lack CCR7 and fail to respond to CCR7 ligands; however, CD16^-^ NK cells express high levels of CCR7, and respond very well to CCR7 ligands. Further, CD56^bright^ NK cells express CD62L, CCR7, CCR5, and CXCR3, which are responsible for their preferential migration into secondary lymphoid organs. Gillard-Bocquet et al. found that CXCR5 and CXCR6 were overexpressed, while the expression of CX3CR1 and S1PR1 was downregulated, relative to non-tumor NK cells (64, 65). A recent study on melanoma revealed that the chemoattractant, chemerin, greatly favors the infiltration of conventional NK cells, T cells, and DCs, but not MDSCs, into tumors, thereby modifying the tumor microenvironment from a tolerogenic to a tumor-suppressive state (26, 66, 67). Matteo Gallazzi et al. found that when co-cultured the healthy donor-derived pNK cells with three different prostate cancer cell lines, together with increased production of pro-inflammatory chemokines/chemokine receptors CXCR4, CXCL8, CXCL12, reduced production of TNF-α, IFN-γ and Granzyme-B (30).

De Andrade et al. found that NK cell frequencies were lower in tumor compared with matching blood samples. They also found that XCL1 and XCL2 were highly expressed among TI-NK cells than blood NK cells, which played the critical role in recruiting DCs to tumor. Besides, TI-NK cells express high level of CCL3, CCL4, CCL4L2 and CCL5, which could bind to CCR5 and other chemokine receptors to recruit T cells and other immune cells. These tumor NK cell populations may thus create distinct microenvironments. NK cells not only kill tumor cells but also recruit key immune cell populations required for protective tumor immunity (46, 68).

## NK Cells Affect Other Immune Cell Effector Functions

Mailloux and colleagues observed that Tregs accumulated within Lewis lung cancer (LLC)-bearing lungs. Further, these Tregs had upregulated CCR4, which can bind chemokines to attract Tregs into LLC-bearing lungs. They also found that LLC-bearing lung tissue secreted elevated levels of CCL22, which can also attract many Tregs to the tumor microenvironment. Surprisingly, the CCL22 was secreted by NK cells, with the phenotype, NK1.1^+^CD11b^dim^CD49b^+^CD122^+^CD27+CD19^+^CD3^−^. Moreover, NK cells and Tregs co-localized in the tumor microenvironment, indicating that Tregs were recruited by NK cells. Thus, if NK cells can stimulate up-regulated CCL22 secretion in the tumor microenvironment, then they may have the unexpected side effect of indirectly contributing to tumor-induced immune suppression, through Treg recruitment (69–71); however, Roy et al. found that NK cells lysed Tregs which expanded in response to an intracellular pathogen, indicating a potential new role for NK cells in maintaining the delicate balance between the regulatory and effector functions of the immune response (72–75).

Russick et al. indicated that some subsets of tumor-infiltrated NK cells express inhibitory markers, including KLRC1 and CTLA4, and that these NK cells may weaken the function of CD8^+^ T cells. When NK cells and DCs are co-cultured, DC maturation is reduced; however, this can be partially reversed by the addition of CTLA4 (19, 76, 77). Neo et al. found that CD73-positive NK cells overexpress multiple alternative immune checkpoint receptors, including LAG3, VISTA, PD1, and PD-L1, and defined this subset of NK cells as regulatory NK cells (78–81). Regulatory NK cells produce IL-10, and/or express the immune checkpoint molecule, CD73, and inhibit autologous CD4^+^ T cell proliferation (29, 82–84).

Erin E. Peterson et al. reported that NK cells and cDC1s engage in intercellular cross-talk integral to initiating and coordinating adaptive immunity to cancer. The NK cell-cDC axis was associated with increased overall survival and anti-PD1 immunotherapy response in patients with metastasis melanoma (85).

## Conclusions

In summary, NK cells are powerful effectors of innate immunity that constitute a first line of defensing against cancer; however, the tumor-microenvironment is highly complex, containing numerous immune-inhibited cells and factors. NK cells can infiltrate primary solid tumors, metastases, and tumor-infiltrated lymph nodes. Tumor-infiltrated NK cells exhibit an altered phenotype, with downregulation of NKp30, NKp80, DNAM-1, and CD16. In addition, expression and secretion of CD107 are impaired. Tumor cells produce soluble molecules, such as IDO, PEG2, TGF-β, and a series of membrane molecules, including PD1, PD-L1, LAG3, TIGIT, and CTLA4. Simultaneously, NK cell metabolism is markedly altered within the tumor-microenvironment, as many molecules, such as FBP1, can directly impair tumor-infiltrated NK cell viability, independent of glycolysis. Further, NK cells can be inhibited by TGF-β produced by Tregs. Tumor-infiltrated NK cells display impaired IFN-γ secretion, which can lead to inefficient DC maturation. NK cells secrete CCL22 to recruit Tregs *via* CCR4 within the tumor and it may intensify the level of immune-inhibition.

Use of cytokines like IL-2, IL-15, IL-12, IL-21 and IL-18 is considered a promising approach to induction of more efficient NK cell activation at tumor sites, while IL-15 and IL-21 can enhance NK cell cytotoxicity (86). Moreover, IL-18-primed NK cells can cooperate with DCs to recruit effector T cells to tumor sites. Immune checkpoint inhibitors, such as lirilumab, which targets KIRs, or monalizumab, which targets NKG2A and the tyrosine kinases inhibitors Imatinib and Sorafenib which aims to enhance the effector function of NK cells by promoting DC-mediated NK-cell activation may improve anticancer responses (87). A better understanding of the roles of tumor-infiltrated NK cells will provide more options for cancer immunotherapy and represents an attractive target to focus on to improve NK cell-based immunotherapies. Also, the comprehensive view of NK cells in the tumor microenvironment will give us inspiration to envisage a future scenario on the research of NK cells and make more favorable clinical outcomes.

## Author Contributions

All authors contributed to the article and approved the submitted version. ZH drafted the manuscript and XX drafted the figures. HW edited/reviewed the article. All authors contributed to the article and approved the submitted version.

## Funding

This work was supported by the key project of the National Natural Science Foundation of China (#81872318 and #81602491).

## Conflict of Interest

The authors declare that the research was conducted in the absence of any commercial or financial relationships that could be construed as a potential conflict of interest.
